# A simplified method to approximate a ROC curve with a Bézier curve to calculate likelihood ratios of quantitative test results

**DOI:** 10.1016/j.mex.2020.100915

**Published:** 2020-05-16

**Authors:** Walter Fierz

**Affiliations:** SVDI, Mittlere Haltenstr. 13, 3625 Heiligenschwendi, Switzerland

**Keywords:** Receiver operating characteristics, Likelihood ratios, Bézier curves

## Abstract

In order to calculate likeli hood ratios (LR) values for quantitative test results, a distribution-independent algorithm based on Bézier curves is proposed. Receiver operating characteristic (ROC) analysis provides the LR as the slope of the tangent to the ROC curve at the point corresponding to the test result.

• Here, we make use of cubic Bézier curves defined by Bernstein polynomials of degree 3.

• A simplified method to adjust a Bézier curve to a ROC curve is presented

• The crucial advantage of this procedure is that Bézier curves are constructed by tangents to the ROC curve, whose slopes immediately provide the LR of a specific point on the curve.

## Graphical abstract

**Figure:**
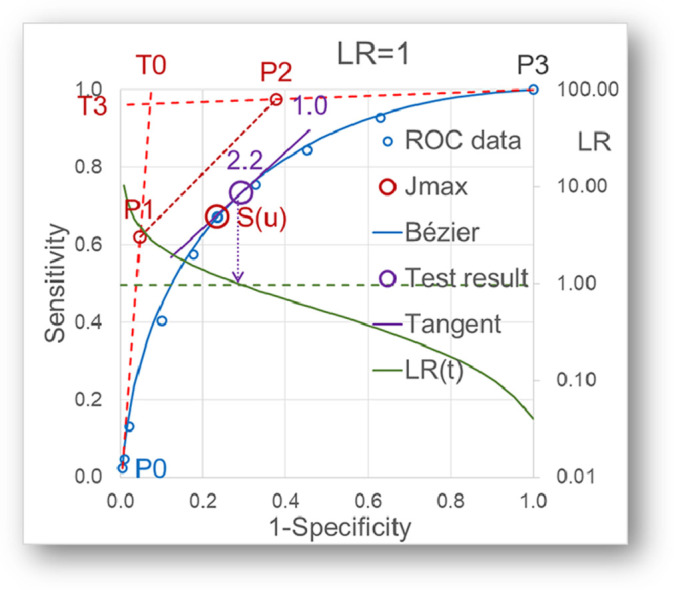

**Table:** Specifications Table

Subject Area	Biochemistry, Genetics and Molecular Biology
More specific subject area	Diagnostic tests
Method name	A simplified method for adjusting Bézier curves to ROC data to calculate likelihood ratios of quantitative test results in medical diagnosis
Name and reference of original method	The application of Bézier curves in ROC analysis W. Fierz, Likelihood ratios of quantitative laboratory results in medical diagnosis: The application of Bézier curves in ROC analysis, PLoS One. 13 (2018) e0192420. https://doi.org/10.1371/journal.pone.0192420.
Resource availability	*If applicable, include links to resources necessary to reproduce the method (*e.g. *data, software, hardware, reagent)*

## Simplified method

### Background: Bayes’ theorem

In medical laboratory diagnostics, the measured quantity in a test gives the physician a measure for estimating the significance of a test result for a particular diagnosis. However, it is left to the physician to assess a quantitative result as to its influence on judging the relevance for a particular disease. In principle, the physician starts with a suspicion for a particular disease. In order to confirm or refute the preliminary suspicion, the physician orders a specific laboratory test and a high result gives a stronger confirmation than a low result.

Formally, in Bayes’ view: pretest odds multiplied by the likelihood ratio (LR) of the quantitative test result give the posttest odds.

## Method

Receiver operating characteristic (ROC) analysis provides the LR as the slope of the tangent to the ROC curve at the point corresponding to the test result. A distribution-independent method has recently been described to calculate these slopes by adjusting Bézier curves to the ROC that are defined by tangents to a curve [1] (Fierz, 2018). The mathematical basis of Bézier curves are Bernstein polynomials of degree n defined by

$$B_{i,n}\left(t\right)=\left(\begin{array}{c} n\\ i \end{array}\right)t^{i}{\left(1-t\right)}^{n-i}\;\text{with}\;t\;\text{ranging}\;\text{from}\;0\;\text{to}\;1.$$

For the purpose here, we make use of cubic Bézier curves defined by

$$B\left(t\right)\;=\;{\left(1-t\right)}^{3}P_{0}+3t{\left(1-t\right)}^{2}P_{1}+3t^{2}\left(1-t\right)P_{2}+t^{3}P_{3}$$

(Fig. 1).

**Fig. 1:**
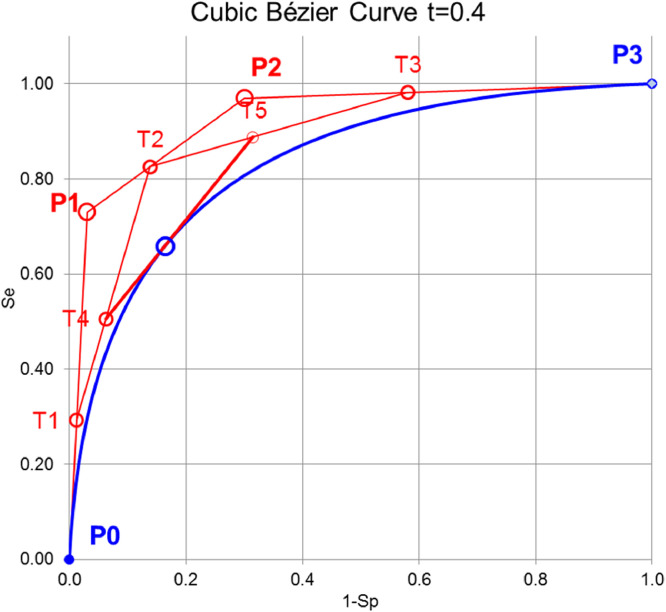
Principle of constructing cubic Bézier curves. First, the lines between the control points P0, P1, P2, and P3 are divided by the ratio t leading to T1, T2, and T3. Second, the lines between T1, T2, and T3 are again divided by the ratio t leading to T4, and T5. Third, the line between T4, and T5 is again divided by the ratio t leading to B(t) on the Bézier curve. The line between T4, and T5 is the tangent to B(t). Pierre Bézier (1910–1999) was a French engineer who developed a method of producing computer-driven curves to be used in the design of automobiles at Renault, which came to be known as Bézier curves.

**Fig. 2:**
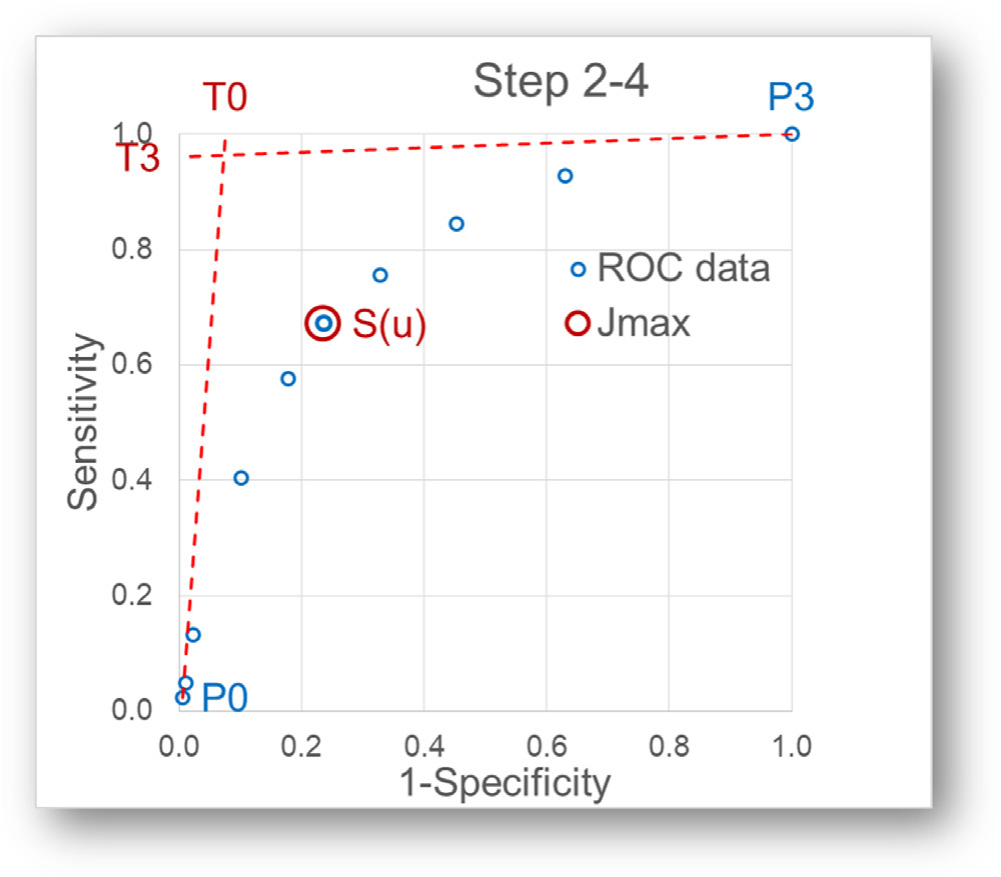
Shape definition of the Bézier curve. Apart from the endpoints P_0_ and P_3_, and the shape point S the shape of the Bézier curve is defined by the two tangents at the endpoints.

Here, a simplified method to adjust a Bézier curve to a ROC curve is presented. The procedure is based on a simplified method for the shape control of cubic Bézier curves [2]. In principle, the shape of the curve is defined by the two endpoints (P_0_ and P_3_) with the corresponding tangents to them and a shape control point B(t)=*S*(u) on the curve (Fig. 2).

## Procedure

Step1: Definition of the variable t of the Bézier curve

The variable t of the Bernstein polynomial has to be introduced and is defined here by t_xy_ = (*x* + *y*)/2 of the empirical ROC points.

Step 2: Selection of the endpoints of the Bézier curve (Fig. 2)

The endpoints of the Bézier curve P_0_=(x_0_, y_0_) and P_3_=(x_3_, y_3_) have to be chosen. Subsequently, the range of t has to be adjusted to the range from 0 to 1 as follows: t_xy_ = ((x-x_0_)/(x_3_-x_0_)+(y-y_0_)/y_3_-y_0_))/2

Step 3: Selection of the shape point of the Bézier curve (Fig. 2)

A shape point S(u) has to be chosen. It is recommended here that from the empirical ROC points the one which is closest to the upper left corner is used. This corresponds to the maximal Youden index (*J*=Se+Sp-1). The t value at that point is denoted u.

Step 4: Selection of tangent vectors at the endpoints of the Bézier curve (Fig. 2)

Apart from the endpoints P_0_ and P_3_, and the shape point S the shape of the Bézier curve is defined by the two tangents at the endpoints:

T_0_ = (g_0_, h_0_) and T_3_ = (g_3_, h_3_) are the tangents vectors at P_0_ and P_3_ respectively.

To start with it is recommended to set

T_0_ = (0, 1) (vertical line) and

T_3_ = (1, 0) (horizontal line) as in an ideal ROC curve.

Later, g_0_ and h_3_ can be adjusted to reach an optimal fit of the Bézier curve to the empirical ROC points.

**Fig. 3:**
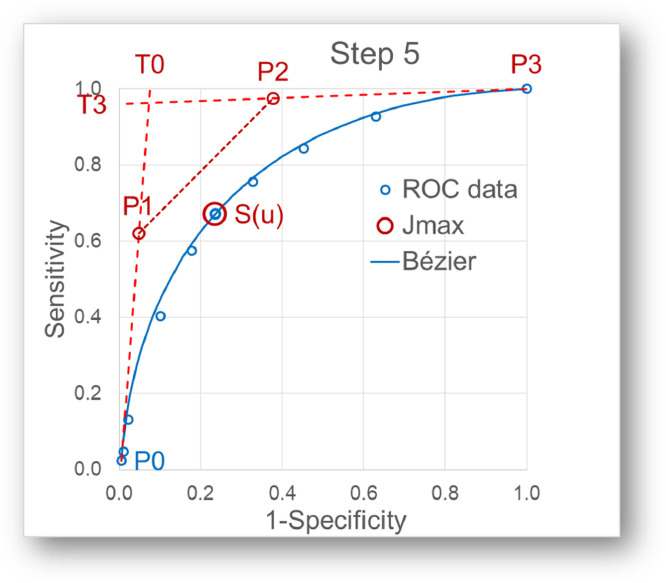
Calculation of P_1_ and P_2_. P_1_ and P_2_ of the Bézier curve can be calculated according to (Yang & Huang, 1993).

Step 5: Calculation of P1 and P2 of the Bézier curve (Fig. 3)

P_1_ and P_2_ of the Bézier curve can be calculated according to (Yang & Huang, 1993) with two shape parameters α and β and the following relations:

$$P_{1}=\left(\alpha /3\right)T_{0}+P_{0},\;\text{and}\;P_{2}=\left(\beta /3\right)T_{3}+P_{3}$$

$$\alpha =\frac{\left|\begin{array}{cc} \left(x_{u}-a*x_{0}-b*x_{3}\right) & g_{3}\\ \left(y_{u}-a*y_{0}-b*y_{3}\right) & h_{3} \end{array}\right|}{u*{\left(1-u\right)}^{2}*\Delta }\text{and}\beta =\frac{\left|\begin{array}{cc} \left(x_{u}-a*x_{0}-b*x_{3}\right) & g_{0}\\ \left(y_{u}-a*y_{0}-b*y_{3}\right) & h_{0} \end{array}\right|}{u^{2}*\left(1-u\right)*\Delta }$$

where the notation | | denotes the determinant operator

$\text{with}\;a={\left(1-u\right)}^{2}*\left(1+2u\right),\;b=u^{2}*\left(3-2u\right)\;\text{and}\;\Delta =\left|\begin{array}{cc} g_{0} & g_{3}\\ h_{0} & h_{3} \end{array}\right|$

**Fig. 4:**
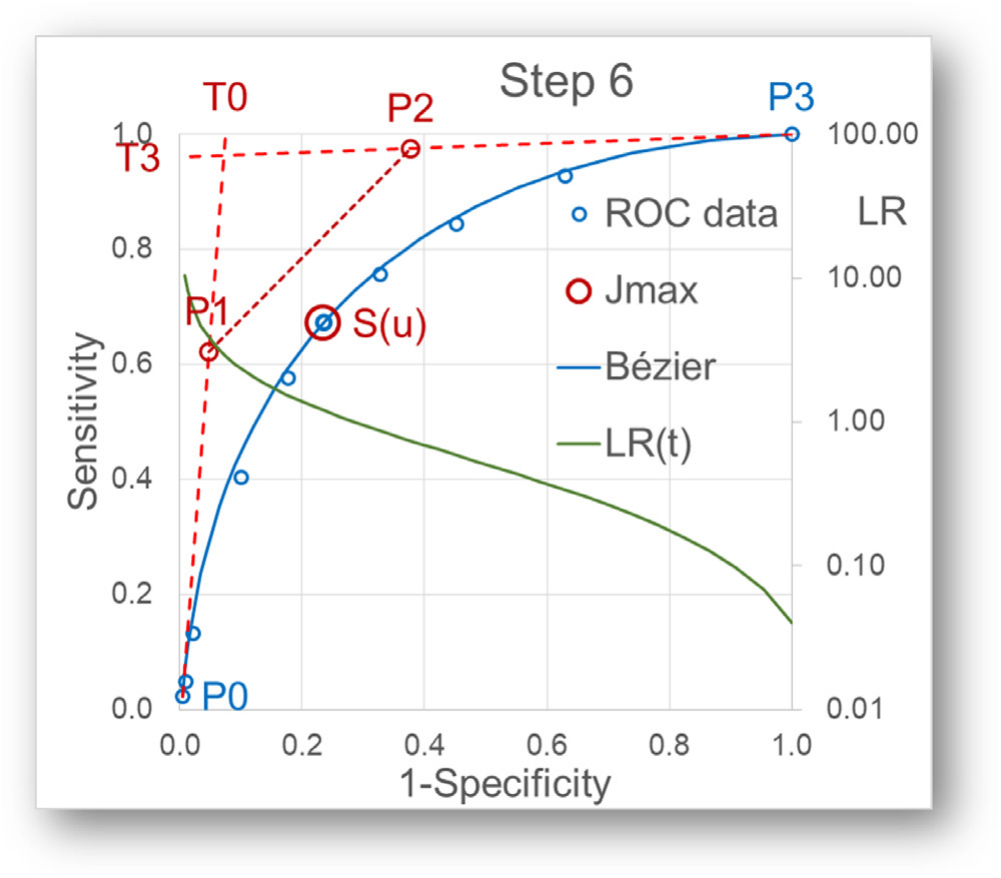
With P_0,1,2,3_ being established in this way, the slopes of the tangents, i.e. the LR(t), can be calculated for all t.

Step 6: Calculation of the tangent slopes (Fig. 4)

With P0,1,2,3 being established in this way, the slopes of the tangents, i.e. the LR(t), can be calculated for all t with

$$LR\left(t\right)=\;\frac{P1y*\left(1-2t\right)-\left(P1y-P2y\right)*\left(2t-3t^{2}\right)+P3y*t^{2}-P0y*{\left(1-t\right)}^{2}\;}{P1x*\left(1-2t\right)-\left(P1x-P2x\right)*\left(2t-3t^{2}\right)+P3x*t^{2}-P0x*{\left(1-t\right)}^{2}}$$

## Validation

To demonstrate the procedure, data from a prosteate-specific antigen (PSA) study are used [3] (Thompson et al., 2005) with the ROC analysis: Gleason Grade >7 (*n* = 250) vs Gleason Grade <7 or No Cancer (*n* = 5325):

“Conclusion (by the authors): There is no cutpoint of PSA with simultaneous high sensitivity and high specificity for monitoring healthy men for prostate cancer, but rather a continuum of prostate cancer risk at all values of PSA.”

**Fig. 5:**
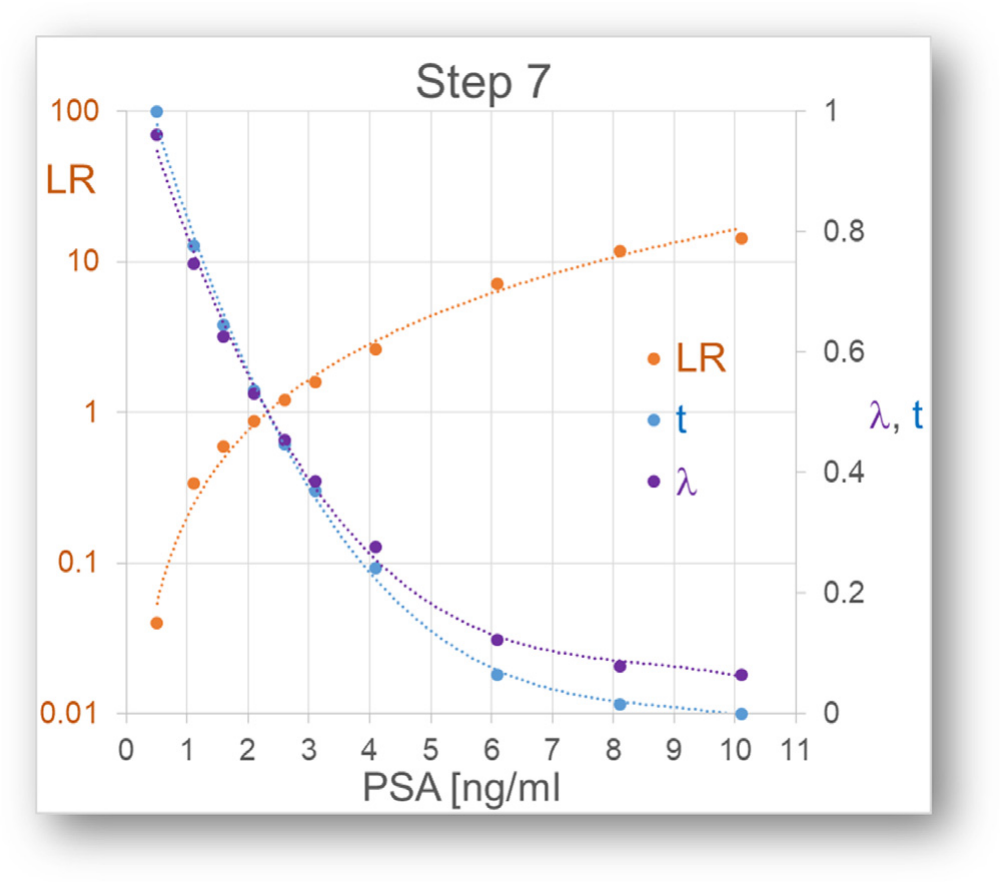
Relation between the quantitative test results and the LR(t)s. Three different calculations according to step 7.

**Fig. 6:**
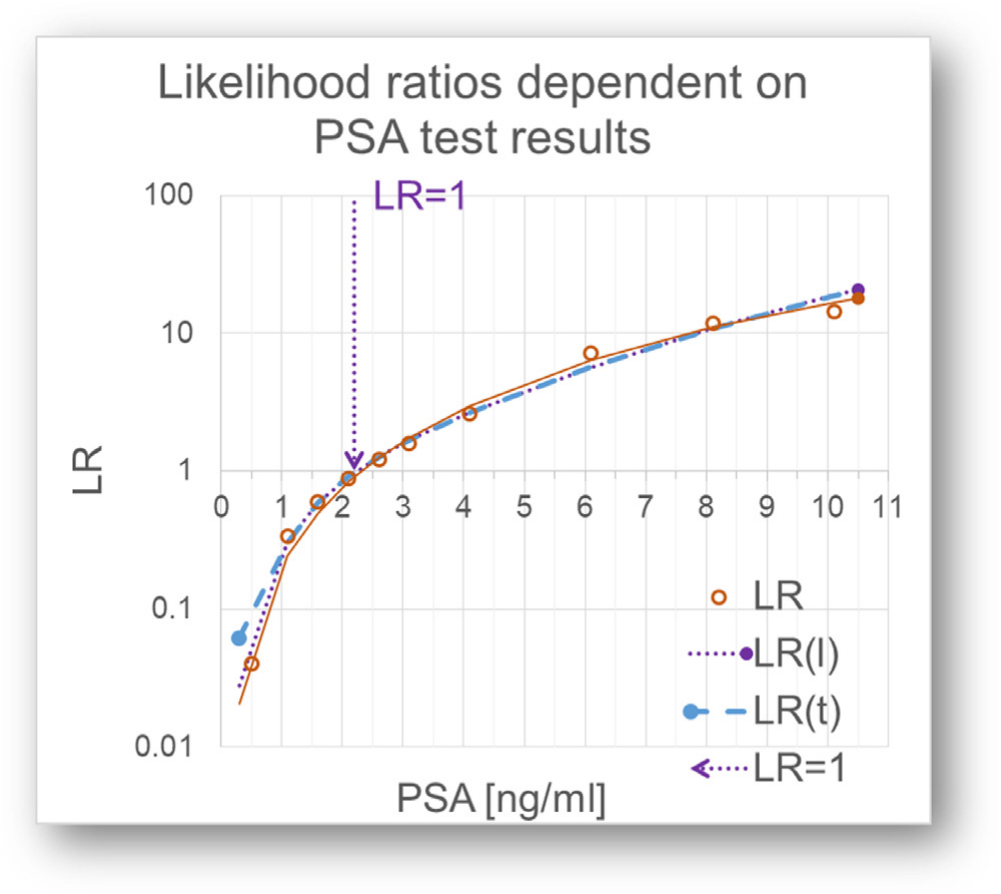
Relation between PSA test results an LRs. LR =1 corresponds to 2.2 ng/ml PSA. Values below 2.2 ng/ml have a LRs <1, values above have LRs >1.

Step 7: Application to PSA test results (Figs. 5 and 6)

The relation between the quantitative test results and their position on the Bézier curve and thereby the LR(t)s has to be established, which of course depends on the test parameter, in this case PSA. This can be done in three ways:

1. Most directly, the LRs of the individual empirical points on the ROC curve are calculated in step 6 and fitted to a curve.
2. More indirectly, a l value based on LR, i.e. *l* = 1/(1+LR) is fitted to the quantitative test values.
3. The t values of the ROC data points are fitted to the test values.

In either way, preferring the method that gives the best fit, the diagnostic LR can be calculated from all quantitative test results.
